# Skin-Derived Stem Cells for Wound Treatment Using Cultured Epidermal Autografts: Clinical Applications and Challenges

**DOI:** 10.1155/2018/4623615

**Published:** 2018-03-25

**Authors:** Inga Brockmann, Juliet Ehrenpfordt, Tabea Sturmheit, Matthias Brandenburger, Charli Kruse, Marietta Zille, Dorothee Rose, Johannes Boltze

**Affiliations:** ^1^Fraunhofer Research Institution for Marine Biotechnology and Cell Technology, Mönkhofer Weg 239a, 23562 Lübeck, Germany; ^2^Insitute of Medical and Marine Biotechnology, University of Lübeck, Ratzeburger Allee 160, 23562 Lübeck, Germany; ^3^Institute for Experimental and Clinical Pharmacology and Toxicology, University of Lübeck, Ratzeburger Allee 160, 23562 Lübeck, Germany

## Abstract

The human skin fulfills important barrier, sensory, and immune functions—all of which contribute significantly to health and organism integrity. Widespread skin damage requires immediate treatment and coverage because massive skin loss fosters the invasion of pathogens, causes critical fluid loss, and may ultimately lead to death. Since the skin is a highly immunocompetent organ, autologous transplants are the only viable approach to permanently close a widespread skin wound. Despite the development of tissue-saving autologous transplantation techniques such as mesh and Meek grafts, treatment options for extensive skin damage remain severely limited. Yet, the skin is also a rich source of stem and progenitor cells. These cells promote wound healing under physiological conditions and are potential sources for tissue engineering approaches aiming to augment transplantable tissue by generating cultured epidermal autografts (CEAs). Here, we review autologous tissue engineering strategies as well as transplantation products based on skin-derived stem cells. We further provide an overview of clinical trial activities in the field and discuss relevant translational and clinical challenges associated with the use of these products.

## 1. Introduction

The skin is among the largest human organs. In addition to its important sensory function, it forms an effective barrier that is pivotal for organism integrity. The skin shields the organism from detrimental environmental influences or infections and maintains a proper fluid balance. It is also one of the most immune-active organs and hosts cellular elements of the innate and adaptive immune system that immediately attack pathogens, should they manage to cross the physical and chemical barrier provided by the epidermis.

Massive and widespread skin damage exceeds the regenerative capacity of the skin, which represents a significant threat to the entire organism and requires timely and effective therapeutic intervention. Moreover, widespread skin lesions often result from burns with thermal damage additionally impairing skin regeneration. Finally, the regenerative capacity of the skin also declines with age, which may necessitate interventions to support wound healing in the elderly.

On the other hand, the skin exhibits a tremendous regenerative potential. Unique stem and progenitor cells reside in the skin and its appendages (e.g., hair bulbs and sweat glands) [1–4] and are sufficient to counter light and moderate skin injury under physiological conditions. These cell populations have been of interest for regenerative medicine approaches since the 1970s to overcome the limitations of conventional skin grafting techniques. A number of therapeutic strategies have already been developed with the potential to thoroughly promote wound healing or replace irreversibly lost skin areas. Consequently, these treatment strategies have been advanced into the clinical arena. In this review, we summarize advances in using skin-derived stem cells and products thereof and monitor the past and recent clinical trial activities in the field. We also identify potential challenges in translation and clinical use that need to be addressed by future research to increase the benefit for patients suffering from complicated skin wounds.

## 2. Conventional Grafting Techniques and Allografts

### 2.1. Autologous Skin Transplantation

Autologous skin grafting techniques can be used to cover large skin defects [5, 6]. For this, skin is taken from another body area of the same patient and widened by cutting and stretching procedures before wound covering (mesh or Meek graft transplantation). Although indispensable for the treatment of severe and large skin wounds, conventional autograft procedures come with some critical limitations. First, autologous skin grafting induces secondary wounds to the patient's skin, which themselves are significant when dealing with larger lesions. Second, massive skin injury resulting from burns, aggressive acidic and alkaline chemicals, or physical stress can cause lesions that are too large to be effectively treated. Third, local blood supply can be impaired, preventing physiological skin regeneration or engraftment. The latter is the case in chronic or diabetic ulcers in the elderly.

### 2.2. Allografts

To overcome some of the problems associated with autologous skin transplantation, skin from other human beings (allografts) or even different species (xenografts) is used. The risk of transmitting a communicable disease cannot be entirely excluded in these approaches [7], but is minimal under modern good manufacturing practice (GMP) conditions and in allografts built from well-characterized cell lines. Premanufactured allografts are cryopreserved and therefore available as off-the-shelf products. Allograft cryopreservation prior to application results in similar clinical outcome in comparison to fresh graft transplantation onto skin ulcers [8]. However, there is no long-term engraftment of major histocompatibility complex (MHC) and blood type-mismatched allografts [9]. Allografts are usually rejected after a mean of 14.5 days, showing signs of acute cutaneous graft versus host disease in histological investigations, but exceptions have also been reported [10]. Rather than engraftment, allografts likely work due to physical wound closure as well as stimulation of endogenous skin regeneration.

Another approach to reduce the immunological response is the use of cell-free biomaterials. One example is Alloderm®, an allogeneic product from a decellularized dermis consisting of collagen fibers and glycosaminoglycans covered by a silicon layer [11]. The product is preferentially applied to deeper wounds. This artificial dermis does not only prevent fluid loss and evaporation from the wound, but also induces cellular proliferation and angiogenesis. These processes sustainably support natural dermis regeneration [12]. Other examples in widespread clinical use are cell-free, xenogenic products such as Matriderm® and Integra® [13, 14].

Such biomaterials are also available in combination with living cells, which support wound healing. Stratagraft® is a product derived from an immortalized human keratinocyte cell line, NIKS (normal immortal keratinocytes), forming a top layer over a dermal fibroblast matrix. Another example is Apligraf®, a collagen matrix containing keratinocytes and fibroblasts of human neonatal foreskin, which shows high proliferative capacity and may have a slightly better immunologic profile. While Stratagraft is primarily employed to treat burn wounds, Apligraf is used to treat leg and diabetic foot ulcers. Epidermal allografts transplanted onto chronic ulcers improve reepithelialization from wound edges and skin appendages, augment granulation tissue, and foster rebuilding of the basement membrane [8, 10, 15].

## 3. Skin-Derived Stem Cells

To overcome immunological problems and the scarce skin availability, cells isolated from small skin biopsies can be propagated in vitro and cultivated on biomaterials to cover skin wounds. These materials are called cultured epidermal autografts (CEAs). CEAs are for instance derived from unpurified epidermal cell cultures that are thought to contain epidermal stem cells.

### 3.1. Epidermal Stem Cells

The first in vitro cultivation of human epidermal keratinocytes forming epidermis-like tissue was reported in 1975 [16]. A mixed cell population containing epidermal stem cells (epi-SCs) was applied in the 1980s and 1990s to treat grade II and III burn victims. Multiple CEAs with a cumulative surface area of up to 1.9 m^2^ (Table 1) were obtained from few small healthy skin biopsies [17]. Although cosmetic results obtained in these early studies were still not optimal, wound healing was significantly improved. Compared to conventionally treated patients, wound surface reduction was accelerated with regenerated skin being more durable and stable [18–20].

CEAs are used in a number of clinical scenarios, but alternative application methods also exist. One approach is to prepare a suspension of autologous epi-SC-derived keratinocytes. This suspension is sprayed onto large burn or chronic wounds showing impaired healing [21]. The procedure is sufficient as a stand-alone treatment of small- and moderately sized, superficial wounds. It significantly improves wound healing and reduces scar formation. The approach is, however, not ideally suited for the treatment of large and/or deeper wounds that usually require additional mesh or Meek grafts. A logistically and cosmetically relevant advantage of the spraying approach is that the wound induced by the skin biopsy can itself be treated with the spray.

### 3.2. Hair Follicle-Derived Epithelial Stem Cells

Hair follicle-derived epithelial stem cells (hf-SCs) are obtained from anagen hair follicles. CEAs generated from these cells are used to treat chronic wounds (predominantly venous ulcers) and have already been applied for more than 20 years [22, 23]. A commercially available autologous product based on hf-SCs called EpiDex® is in clinical use since 2004. About 4 weeks are required to cultivate numerous small EpiDex discs of about 1 cm in diameter (Table 1). Interestingly, donor (patient) age has no influence on cell proliferation [24] and the overall efficacy of the approach. EpiDex is typically applied to small and moderately sized chronic wounds exhibiting granulation but not reepithelialization. A long-term study performed between 2004 and 2008 revealed that EpiDex treatment induced complete wound healing in 3 out of 4 cases within a 9-month surveillance period [24].

## 4. Clinical Use of CEAs

The first transplantations of human CEAs in a clinical case series were conducted in 1980 [25]. Two patients suffering from partial and full-thickness burns on 80% and 40% of their body surface area, respectively, were treated with split-thickness skin grafts and epi-SC-derived CEAs. Direct comparison between the two transplantation methods did not indicate differences in graft contraction or fragility. Although there were differences compared to what is found in normal skin, regenerating skin spreading in from the wound edges resulted in similar tissue histology [25].

The durability of CEAs was further demonstrated by two cases, in which children suffering burns continued to live for at least 20 years after being transplanted with cultured autologous equivalents in the early 1980s [26, 27]. Moreover, a female burn victim experienced normal pregnancy despite the previous abdominal transplantation of CEAs grown on a fibrin matrix [28]. This demonstrates that CEAs do not only provide tissue replacement and homeostasis, but can also grow and adapt to mechanical stress, a decisive feature for improving quality of life after transplantation, particularly in young patients.

Based on the early successes in burn victims, a wide spectrum of possible applications other than burns was assessed over the years. For instance, skin defects following pyoderma gangrenosum [29], excision of congenital nevi [30], separation of conjoined twins resulting in large wounds on the left side of the thoracic and abdominal walls [31] as well as in vitiligo [32] and chronic leg ulcers [33] were treated with CEAs. Furthermore, an autologous method for junctional epidermolysis bullosa (JEB) treatment was reported in 2006. A retroviral vector expressing LAMB3 cDNA was used to manufacture genetically modified CEAs for a JEB patient. Transplantation of these grafts resulted in adherent and completely functional epidermis. No complications were reported during the 1-year follow-up period [34]. Lenti- and retroviral vectors as well as a ϕC31 integrase-based gene correction method were also investigated for (recessive) dystrophic epidermolysis bullosa [35–38]. Transfected epi-SC-derived CEAs exhibited regular distribution of type VII collagen as well as normal epidermal differentiation and morphology for at least 12 months in a xenogeneic (human-mouse) transplantation model [38]. A clinical study using this retroviral vector is currently ongoing (NCT01263379). In a very recent single-case clinical intervention, a pediatric patient suffering from severe and life-threatening JEB was treated with genetically corrected epidermal sheets derived from a 4 cm^2^ autologous skin biopsy [39]. Outcome was highly encouraging and the treatment concept has the potential to be widely applied.

Currently, there are only few commercial CEAs available on the market. Among these are Epicel® (Vericel, USA), ReCell® (Avita Medical, UK), and MySkin® (Regenerys, UK), the latter being an unlicensed medicinal product. Epicel is prepared as a sheet and MySkin as a single-cell suspension. Both are produced under GMP conditions. ReCell is not a cell product, but a device for preparing a cell suspension from a single-skin biopsy directly in the clinic. The suspension is sprayed on to the lesioned area.

### 4.1. Benefits of CEAs

The main advantage of CEAs compared to conventional skin autografts for the treatment of complicated or chronic wounds is that it does not require a second skin wound, which itself can be prone to complications such as pain, infection, retarded healing, and scar formation [40]. For this purpose, the scalp is an advantageous skin source as it contains abundant hair follicles (leading to better epithelialization) and any potential scars can be covered relatively easily. The biopsy process is well tolerated and avoids larger secondary wounds [38]. Epi-SCs are usually obtained via skin biopsy under local anesthesia, whereas hf-SCs are acquired by hair follicle plucking without the need for anesthesia.

After grafting, patients need to be immobilized only shortly, if at all [23]. One reason for this is that CEAs lead to slightly thinner but more elastic epidermis formation. Moreover, application of CEAs reduces wound contraction as compared to conventional approaches, and hypertrophic scar formation is less frequent [41]. Wounds display only minimal contraction, but maintain excellent tissue flexibility when treated with epi-SCs cultivated in fibrinogen-derived fibrin glue, made of fibrinogen, fibronectin, and factor XIII. Hence, this approach is particularly feasible for areas under intensive and/or complex mechanical stress [28] such as the eyelid, fingers, or toes. Furthermore, epi-SCs cultivated in the fibrinogen matrix provided by the glue form a stable cell layer, which is easy to handle during transplantation and shows excellent adhesion to the wound surface [42]. The combination with allografts is also possible, as the top silicon layer of Alloderm can be removed and replaced by a CEA [43].

### 4.2. Overview of Clinical Trial Activities

**Figure 1:**
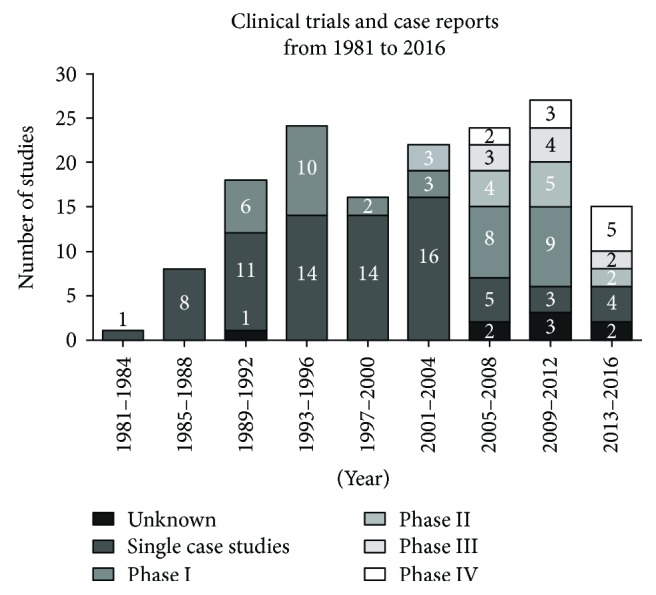
Number and type of clinical trials for stem cell-based skin regeneration since 1980. Single-case studies or small series of single cases were the main clinical activities reported before 1989. Subsequently, the number of phase I trials (safety) increased, reaching its first peak in the mid-1990s, followed by phase II studies (safety and secondary efficacy endpoints) since the early 2000s. Large, late-stage clinical phase III (efficacy) and phase IV (surveillance of products on market) studies were reported since 2005, which was accompanied by a drop in single-case reports. Remarkably, no additional phase I clinical trial has been launched since 2013. Only studies reported in http://pubmed.com and/or listed on http://clinicaltrials.gov were included in this analysis. The search was restricted to studies reported between 1981 and 2016.

To achieve an overview on recent activities in the application of CEAs, we performed a literature and database search on “http://pubmed.gov/” and “http://clinicaltrials.gov/”. Search terms are provided in Supplementary Table 1. Our search revealed 155 clinical trials and single-case reports being performed and published between 1981 and 2016. Skin-derived stem cells and related products were used in both clinical trials and single-case treatments (Figure 1). Early clinical activities reported in the 1980s and 1990s were primarily single-case reports. Phase I (safety) studies started to emerge around 1990. Studies including secondary or primary efficacy endpoints (phases II to IV) were conducted since 2005, indicating the step-wise development and progression of tested approaches and products.

Of note, new phase I clinical trials have not been launched since 2013, and a considerable proportion of clinical studies were not completed as planned. In particular, only 12 out of 40 studies (30%) listed on “http://clinicaltrials.gov/” have been completed as originally intended. Another 13 (32.5%) studies are still active, with 11 studies (27.5%) currently recruiting patients. A total number of 15 studies (37.5%) were terminated, withdrawn, or have an unknown status. This means that at least about one-third of all studies were not performed as planned. Moreover, some of the completed studies ended without recruiting the prespecified patient numbers; hence, it is unclear whether the remaining 11 studies currently enrolling patients can be completed as planned.

The shortage of studies in the last years may indicate the potential challenges hampering clinical implementation of stem cell-based skin regeneration approaches. In the following paragraphs, we will highlight unmet research needs reflecting such challenges in skin wound treatment using CEAs.

## 5. Unmet Research Needs and Challenges

### 5.1. Preclinical Research

Results from in vivo models can usually not be completely transferred to the clinical situation in experimental dermatology and wound healing. This is partially due to interspecies differences. Compared to murine skin, which represents the most popular in vivo model in experimental dermatology, for instance, humans have thicker epidermal and dermal layers containing more cells. Furthermore, human skin contains fewer but larger hair follicles while the interfollicular epidermis is wider-spaced [44, 45]. Human skin also exhibits rete ridges and a number of basic immune system differences [44–46]. For example, surface proteins of human and mouse hf-SCs are not congruent [47].

Skin contraction is an important feature in rodent wound healing, but is not observed in humans and thus may lead to an overestimation of treatment effects as compared to human patients [48]. A number of measures have been developed to counter these effects in rodent models, including the use of chambers or polypropylene rings and tetanized meshes that are transplanted into the skin defect to impair wound contraction [49–51]. Suturing the wound edges to underlying tissue is also performed to stabilize the defect [52]. Although these techniques are effective in preventing skin contraction in rodents, they interfere with the wound healing process and therefore cannot fully compensate for interspecies biases in preclinical in vivo wound healing studies. One option is to use large animal models for confirmative studies in the field. Pig skin is more similar to human skin and porcine models may provide valuable advantages in translational research [53]. However, the overall number of large animal studies on wound healing, even when including neighboring areas of skin regeneration research, is limited to only a few key publications [54–56]. This may partially be due to the overall higher costs and efforts of large animal research [57]. Nevertheless, the use of more advanced in vivo or, alternatively, ex vivo wound healing models more adequately representing human skin pathophysiology is encouraged.

### 5.2. CEA Production and Treatment Costs

One of the major disadvantages of CEA is its relatively long production time of 3 to 4 weeks (Table 1), which is problematic when treating large-sized and complicated skin wounds. To solve this problem, a two-step wound treatment approach can be applied. Initially, an allograft is transplanted for wound coverage as an intermediate outer barrier, gaining the required time for the generation of patient-specific CEA autografts, which are subsequently transplanted.

The relatively high costs associated with the use of CEAs present another challenge preventing widespread application. In the late 1990s, treating 1% of the surface area of the body after burns was estimated to cost US$600 for CEAs cultured on fibrin and US$1350 for autografts cultured on special plastics [58], and costs may have further increased since then. The cost per square centimeter with definitive engraftment and wound closure in children was calculated to be around US$6520. A comparable treatment in adults is even more expensive (US$13,000), with a reported definitive engraftment rate of just 4.6%, questioning the cost-effectiveness of such treatments in adults [59]. Daily care expenses for a burn victim treated with an epidermal autograft amount to US$4500, clearly exceeding the costs of a conventional treatment (US$3500) [59]. High treatment costs, in part being caused by the indispensable, but labor-intensive and expensive manual GMP production, are therefore a relevant aspect at least partially explaining why stem cell-derived CEAs are not yet routinely and widely applied. Manual GMP production gives a relatively small product output [28]. The overall high production costs can only be covered if there is a high and continuous request for CEAs, but the demand often remains behind expectations. This may explain frequently observed bankruptcy in skin regeneration companies [60]. Step-wise implementation of automation technology into the production chain may help to lower overall production costs. An alternative would be the utilization of simplified, on-site cell isolation procedures from autologous skin or subdermal fat tissue, but it needs to be shown that the therapeutic outcome in the clinics compares to the promising preclinical data [61] and results obtained by CEAs currently in use.

On the other hand, a cost-effectiveness study in children with massive burns, conducted in the late 1990s, revealed that burn sites treated with cultured grafts show less extensive scar formation compared to those treated with mesh grafts [62]. This may warrant the treatment particularly in younger patients, even though treatment with CEAs is associated with longer hospital stays. In addition, patients treated with CEAs had to undergo more reconstructive procedures during the first two years after treatment [62]. Although good clinical experience was made [63], a recent and detailed cost calculation for EpiDex in the treatment of leg ulcers still reported significant treatment costs. However, the direct comparison of EpiDex and split-thickness skin grafting for a single average treatment resulted in a better cost-effectiveness in favor of EpiDex. The price for one EpiDex disc with a diameter of 1 cm amounts to US$480 and, depending on the wound surface area, between 6 and 12 discs are required per treatment [24].

Nevertheless, Epidex disappeared from the market after the final supplier, Euroderm, went bankrupt in 2014. The original manufacturer, Modex Therapeutics, fused with IsoTis, a company from the Netherlands in 2002. The wound care portfolio was sold to DFB Pharmaceuticals (USA) one year later. However, there are no wound care products on the official DFB pharmaceuticals webpage as of 2017.

### 5.3. Challenges and Complications in Clinical Use

Frequent complications in burn patients after epidermal autograft treatment comprise high failure rates, abundant scarring, and fragile skin coverage [64]. Clinically, fragile skin coverage manifests in blistering and may be caused by a delay in dermoepidermal junction formation [59]. However, the rate of major complications is similar between patients treated with CEAs or conventional grafting techniques [59].

A major and thus far unsolved problem is the variable engraftment rate. Engraftment can be complicated by wound infection [25, 42, 65] and depends on size and depth of the wound. Age may also affect the engraftment rate, with some studies reporting younger patients showing higher engraftment rates [65, 66]. Treated wounds require careful primary and secondary wound coverage to prevent graft displacement or damage. Moreover, CEAs can show signs of hyperkeratosis [65].

#### 5.3.1. Infections

Concurrent infections were speculated to be a main reason for graft failure since the first application of CEAs [25]. Indeed, wound infection was the most common adverse reaction in a trial assessing the commercially available product EpiDex for ulcers treatment [24]. These infections were so far treated with systemic antibiotics or topical antimicrobials and antiseptics (for details see Drug Interactions).

A successfully treated infection does not impair subsequent epithelialization [67], but some bacterial species cause complications or even graft failure requiring regrafting. For instance, *Pseudomonas aeruginosa* destroys split-thickness skin grafts as well as epidermal autografts [30, 68]. Similarly, *Staphylococcus haemolyticus* induces graft destruction and concurrent bleeding at the treatment location, which in some cases may even lead to death [28].

Moreover, graft failure can also be caused by the “melting graft-wound syndrome.” This syndrome is characterized by increasing loss of epithelium from a healed burn wound, a formerly well-taken graft or donor site, lack of signs of systemic infection, and absence of inflammation or cellulitis of the surrounding skin [69]. *Streptococcus* species may be responsible for epithelial cell loss, but cases without bacterial manifestation have also been reported [63]. Further investigation of this phenomenon is therefore needed [70].

A carefully prepared wound bed and effective infection control are important prerequisites for proper engraftment [58], reflected in the application notes of commercially available products. For instance, Apligraf must not be used on infected ulcers [71]. One of the causes making CEAs vulnerable for infections is inferior perfusion of the graft. Hence, strategies enhancing vascularization and augmenting supply as well as immunologic defense capabilities in the wound area upon transplantation need to be developed [49].

#### 5.3.2. Drug Interactions

The potential interaction between drugs commonly applied to patients with skin deficits and grafted material is clinically relevant. As reviewed above, infections are a common problem in CEA application and can damage or destroy the graft.

Keratinocytes in the upper graft layers are the most important cell population for graft functionality. These cells shield underlying tissue from environmental influences. Hence, several topical antimicrobial substances used to fight common infections in patients have been tested on cultured keratinocytes to assess potential toxicity on transplanted grafts. Aminoglycosides, macrolides/lincosamides, glycopeptides, and polypeptides exert cytotoxic effects on keratinocytes, with cytotoxicity increasing in the listed order. In addition, nonsynthetic antibiotic substances are less cytotoxic than synthetic or antifungal substances [72].

Table 2 summarizes the main results of toxicity studies using keratinocytes. The least toxic antibiotics with respect to clinically applied dosages can be ranked as follows (increasing order of toxicity): neomycin, clindamycin, framycetin, erythromycin, and gentamycin. These substances are therefore recommended for use with epidermal grafts including CEAs. Toxicity of topically applicable antiseptics is (in that order, highest toxicity first) silver sulfadiazine, silver nitrate solution, cerium-silver-sulfadiazine, and silver nitrate plus chlorhexidine.

An interesting point of ongoing discussion is the systemic intake of topical agents and related side effects. This may be relevant when antibiotics are applied to large body surface areas, for instance, after severe burn injury. Only a few investigations have addressed this problem so far. For example, the gentamycin concentration in the blood after topical application on full-thickness wounds was clearly below the recommended maximum level in a pig study, reducing safety concerns of topical application on moderately sized skin wounds [73].

#### 5.3.3. Ulceration and Malignant Degeneration

Treatment with CEAs can also lead to more severe complications in single cases. For instance, several secondary lesions and ulcerations of unknown origin were observed in a burn patient following extensive epi-SC-derived autograft transplantation. Even an invasive squamous cell carcinoma representing a potential malignant transformation was observed 13.5 years after the initial treatment, followed by another seven carcinomas over a time period of 10 years [74, 75]. The use of cholera toxin or isoproterenol during the in vitro cultivation of epi-SCs may be one reason for this transformation. It can, however, not be concluded that carcinoma induction is an adverse event that can solely be traced back to the cultivation and transplantation of CEAs, since malignant transformations can also emerge from burn scars over time [74–76]. These observations illustrate the necessity for continuous and thorough follow-up of transplant patients [77].

#### 5.3.4. Cosmetic Aspects

All currently available grafts fail to regenerate skin appendices such as sweat or sebaceous glands and hair. Particularly, lack of the latter can lead to suboptimal cosmetic results even if wound healing was sufficient. A single case was reported of a large (10 × 8 cm) cranial full-thickness burn wound treated with a tissue-engineered dermal template [67]. Hair follicles were micrografted into the template and, being a stem cell source, contributed to complete reepithelialization within just 37 days. However, only very minor regrowth of hair was observed one year after treatment.

Very recent preclinical studies reported the possibility to generate fully functional hair follicles by stem cell manipulation [78]. Similar results were reported for protocols using induced pluripotent stem cells [79]. Such techniques may in the future be used to promote hair regrowth on transplanted skin, but require further refinement for application in patients.

## 6. Summary

Autologous and allogeneic skin grafts cultured from skin-derived stem cells can efficiently support wound healing and are a valuable addition to conventional skin grafting approaches. The combination with biomaterials can augment stability and functionality of the transplants. A number of successful clinical trials have been completed, and bioengineered skin grafts are available as therapeutic products. Nevertheless, the use of these products is still limited. First, they are still expensive to manufacture, limiting widespread applicability. Automation of graft production and application may help to overcome this limitation. Second, treatment results are often limited since skin appendices (glands, hair) are usually not regenerated. Advanced stem cell and tissue engineering approaches may provide solutions for this in the future. Third, results of in vivo (rodent) studies are only partially comparable to the patient situation, warranting the development of novel models including large animal experiments to achieve better comparability. Moreover, potential interactions between topical and systemic drugs and the stem cell-derived graft should be investigated in more detail. Altogether, this will augment the therapeutic value and clinical applicability of skin grafts.

## Figures and Tables

**Table 1:** Comparison between epi-SC- and hf-SC-derived CEAs.

Aspect	epi-SCs	hf-SCs	References
Reported donor age	0 to 59 years	63 to 91 years	[22, 65, 80]
Cultivation period	3 to 4 weeks	4 weeks	[25, 66, 81]
Material required	Skin biopsy (3 cm^2^)	40 to 350 anagen hair follicles	[28, 66, 82]
CEA surface	0.8 cm^2^ single-CEA surface or larger up to 1.9 m^2^ in total can be generated for one patient	0.8 cm^2^ single-CEA surface	[17, 22, 23]
Engraftment rate	70%, high variability	80 to 90%	[18, 19, 82]

**Table 2:** Experiences with drug interactions on skin cells.

Chemical name	Brand name	Toxicity	References
Fusidic acid, tetracycline, virginiamycin	Diverse	High toxicities at clinically applied doses	[72]
Gentamycin	Diverse	Favorable safety profile at low concentrations	[73, 83]
		Impaired cell migration and proliferation at higher concentrations (0.1 to 1.0 mg/mL), thus the clinically applied dose in topical preparations (about 0.1% or 1 mg/g) may impair cell function	
Mafenide	Sulfamylon®	Cytotoxic even at lower local concentrations, therefore not suitable as a topical agent	[83]
Phenoxyethanol	Diverse	Promising alternative to antiseptic solutions	[72]
Polymyxin B	Diverse	Dose-dependent detrimental effects	[83]
Polymyxin B sulfate in combination with bacitracin	Polysporin®	Impaired proliferation at higher concentrations with the main effect being mediated by polymyxin B sulfate	[83]
Polymyxin B sulfate in combination with neomycin sulfate	Neosporin®	Much more favorable safety profile, containing far less polymyxin B but exerting excellent antimicrobiotic effects, discussed as a well-suited topical antibiotic	[83]
Povidone-iodine	Diverse	Toxicity depends on the presence of serum (higher without)	[72]

## Supplementary Materials

Supplementary Materials Supplementary Table 1: search terms included in the trial search. It contains all search terms and combinations that have been applied for the online research regarding relevant publications of clinical applications in the field. Online research was performed at the “http://clinicaltrials.gov/” and “http://pubmed.com/” data bases. Clinical studies and case reports/series have been included.
