# Supplementary material for: Skin-Derived Stem Cells for Wound Treatment Using Cultured Epidermal Autografts: Clinical Applications and Challenges
Source: Stem Cells Int. 2018 Mar 25;2018:4623615. doi: 10.1155/2018/4623615 (PMC5889868; doi:10.1155/2018/4623615)
Supplement: Supplementary Materials — Supplementary Table 1: search terms included in the trial search. It contains all search terms and combinations that have been applied for the online research regarding relevant publications of clinical applications in the field. Online research was performed at the “http://clinicaltrials.gov/” and “http://pubmed.com/” data bases. Clinical studies and case reports/series have been included. [file 4623615.f1.pdf]

Supplementary Table 1. Search terms included in the trial search.

<table>
<tr><th>Search terms used</th><th colspan="2">Search terms and combinations revealing results</th></tr>
<tr><td rowspan="3">Apligraf
autologous epidermal cells
autologous keratinocytes
cultured autologous keratinocytes
cultured epidermal autograft
cultured epithelial autograft
epidermal autograft
epidermal equivalent
epidermal sheet
hair follicle
keratinocytes
OrCel
skin
Stratagraft
wound</td><td>at “clinicaltrials.gov”</td><td>Apligraf
autologous keratinocytes
epidermal sheet
keratinocytes
OrCel
Stratagraft</td></tr>
<tr><td>at “pubmed.com”
(case reports)</td><td>cultured autologous keratinocytes
cultured epidermal autograft
cultured epithelial autograft
epidermal equivalent
hair follicle AND wound</td></tr>
<tr><td>at “pubmed.com”
(clinical trials)</td><td>autologous epidermal cells
cultured autologous keratinocytes
cultured epidermal autograft
cultured epithelial autograft
epidermal autograft
epidermal equivalent AND wound
hair follicle AND wound</td></tr>
</table>
